# Superior semicircular canal dehiscence isolation by transmastoid two-point canal plugging with preservation of the vestibulo-ocular reflex

**DOI:** 10.1007/s00106-024-01533-9

**Published:** 2024-12-11

**Authors:** Ingmar Seiwerth, Julia Dlugaiczyk, Frank Schmäl, Torsten Rahne, Sabrina Kösling, Stefan K. Plontke

**Affiliations:** 1 Department of Otorhinolaryngology, Head and Neck Surgery, Martin Luther University Halle-Wittenberg, University Hospital Halle (Saale), Ernst-Grube-Str. 40, 06120 Halle (Saale), Germany; 2 Department of Otorhinolaryngology, Head and Neck Surgery & Interdisciplinary Center for Vertigo, Balance and Ocular Motor Disorders, University Hospital Zurich (USZ), University of Zurich (UZH), Zurich, Switzerland; 3 Dizziness outpatient clinic of the ENT Center Münster/Greven, Greven, Germany; 4 Department of Radiology, Martin Luther University Halle-Wittenberg, University Hospital Halle, Halle-Wittenberg, Germany

**Keywords:** Superior semicircular canal dehiscence, Vestibular evoked myogenic potentials, Head impulse test, Third window syndrome, Bogengangdehiszenz, Vestibulär evozierte myogene Potentiale, Kopfimpulstest, Syndrom des dritten Fensters

## Abstract

This article describes the surgical treatment of superior semicircular canal dehiscence syndrome (SCDS) by isolating the dehiscence using transmastoid two-point canal plugging while preserving the high-frequency vestibulo-ocular reflex (VOR) of the affected semicircular canal. The superior semicircular canal is opened via a transmastoid approach anterior (as far from the ampulla as possible) and posterior to the dehiscence and then plugged with connective tissue and bone dust. In two clinical exemplary cases, vestibular testing showed that the VOR measured by video head impulse (vHIT) test was preserved (patient 1: gain preoperative 0.7, long-term postoperative 0.75; patient 2: gain preoperative 0.64, long-term postoperative 0.79; reduction of corrective saccades in each case) with a simultaneous reduction in pathologically increased amplitudes of vestibular evoked myogenic potentials (VEMPs) and a significant improvement in clinical symptoms with almost complete freedom from symptoms. One possible explanation for preservation of the high-frequency VOR of the superior semicircular canal would be the deformability of the endolymphatic space described at high stimulation frequencies, which can lead to endolymph movements in the area of the ampulla with deflection of the cupula despite blockage of the semicircular canal.

**Video online:**

The online version of this article contains a video. The article and the video are available online (10.1007/s00106-024-01533-9). The video can be found in the article back matter as “Electronic Supplementary Material”.

## Abstract (de)

Im vorliegenden Beitrag wird die operative Therapie des Dehiszenzsyndroms des oberen Bogengangs (SCDS) durch Isolation der Dehiszenz mittels transmastoidaler 2‑Punkt-Verblockung des Bogengangs unter Erhalt des vestibulookulären Reflexes (VOR) des betroffenen Bogengangs beschrieben. Dabei wird der obere Bogengang über einen transmastoidalen Zugang anterior (möglichst ampullenfern) und posterior der Dehiszenz punktförmig eröffnet und mit Bindegewebe und Knochenmehl verblockt. Die Funktionsdiagnostik zeigte an zwei klinischen Beispielfällen einen Erhalt des mittels Video-Kopfimpulstests gemessenen VOR (Pat. 1: Gain präoperativ 0,7; langzeit-postoperativ 0,75; Pat. 2: Gain präoperativ 0,64, langzeit-postoperativ 0,79; jeweils Reduktion der Korrektursakkaden) bei gleichzeitiger Reduktion pathologisch erhöhter Amplituden vestibulär evozierter myogener Potenziale (VEMP) und einer deutlichen Verbesserung der klinischen Symptomatik mit weitestgehender Beschwerdefreiheit. Ein möglicher Erklärungsansatz für den Erhalt des VOR des oberen Bogengangs wäre die insbesondere bei hohen Reizfrequenzen beschriebene Verformbarkeit des Endolymphschlauchs, wodurch es trotz Verblockung des Bogengangs zu Endolymphbewegungen im Bereich der Ampulle mit Auslenkung der Cupula kommen kann.

## Superior semicircular canal dehiscence syndrome

Superior semicircular canal dehiscence syndrome (SCDS) was first described by Minor et al. [14] in 1998. It is characterized by a bony defect of the superior semicircular canal with characteristic symptoms and findings, such as noise- and pressure-induced vertigo and nystagmus (Tullio phenomenon), autophony, improved bone conduction hearing thresholds, and conductive hearing loss. Pathophysiologically, the symptoms are explained by a “third window”. In addition to the oval and round windows, the dehiscence is another window through which sound can enter and exit the inner ear. If sound is transmitted to the ampulla of the semicircular canal via the third window in a nonphysiological way, this can lead to vertigo, while a loss of air-conducted sound energy via the dehiscence may induce conductive hearing loss on the affected side with simultaneously improved bone conduction hearing thresholds (“inner ear-related [pseudo-]conductive hearing loss” [6, 12, 20]).

The diagnosis of SCDS is made according to the criteria of the International Classification of Vestibular Disorders (ICVD) and requires all of the following criteria to be met:

1. At least one of the following symptoms: bone conduction hyperacusis, sound- or pressure-induced vertigo/oscillopsia, pulsatile tinnitus
2. At least one of the following signs or diagnostic test results: sound- or pressure-induced nystagmus, negative bone conduction hearing thresholds, increased vestibular evoked myogenic potential (VEMP) responses
3. Evidence of dehiscence on high-resolution computed tomography (HRCT)
4. No better explanation through another vestibular disorder or disease [21]

As there are no known effective drug therapies for SCDS, the only treatment option is surgery if the patient reports relevant distress and impairment. The aim of surgery is to reduce or eliminate the phenomenon of the third mobile window. Effective SCDS surgery must adequately and permanently seal the dehiscence in a fluid-tight manner against nonphysiological pathways of pressure and sound waves through the inner ear. This is usually achieved by “plugging” the semicircular canal in the area of the dehiscence via a transtemporal (middle cranial fossa) or via a transmastoid approach. A “resurfacing” or “capping” technique (restoration of the bony boundary of the semicircular canal) or a combination of different techniques can also be used with both surgical approaches [6, 20, 22].

Transmastoid two-point plugging of the superior semicircular canal to eliminate or isolate the dehiscence is a microscopically or endoscopically assisted surgical procedure [1, 2]. As with all surgical interventions, functional impairments in the affected sensory organs should be avoided or kept to a minimum. This article describes the surgical treatment of SCDS via isolation of the dehiscence using transmastoid two-point plugging of the semicircular canal while preserving the high-frequency vestibulo-ocular reflex (VOR) measured by video head impulse testing (vHIT) using two exemplary cases.

## Surgical technique

**Fig. 1:**
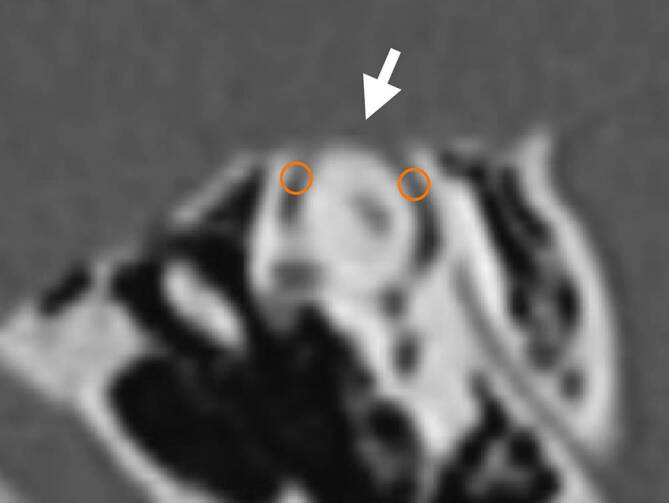
Dehiscence (*arrow*) of the superior semicircular canal on the right in an oblique coronal reconstruction (parallel to the superior semicircular canal plane, previously referred to as “Pöschl plane”) of a thin-slice spiral computed tomography data set of the temporal bone (patient 2). The two circles mark the points for opening the semicircular canal and isolating the dehiscence

**Fig. 2:**
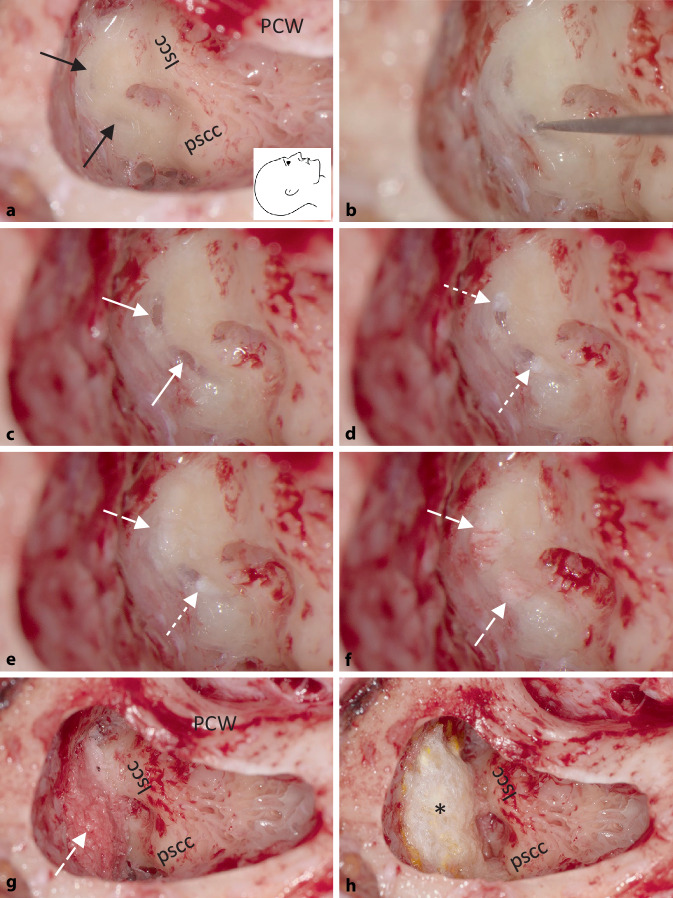
Surgical steps during isolation of the superior semicircular canal dehiscence using transmastoid two-point plugging (right ear). **a** Simple mastoidectomy and “blue lining” (“gray lining”) of the superior semicircular canal (→). **b** Further thinning of the bone anterior (as far as possible from the ampulla) and posterior to the dehiscence and lifting off the remaining bone chips with a 0.2-mm stapes footplate hook. **c** Selectively opened semicircular canal anterior and posterior to the dehiscence. **d–f** Insertion of wet connective tissue, first anterior to the dehiscence in the direction of the ampulla and then posterior to the dehiscence in the direction of the common crus and then of bone dust in the direction of the dehiscence. **g, h** Covering of the semicircular canal and small areas of exposed dura with bone dust and TachoSil® (*). White arrows indicate opened semicircular canal, dotted arrows indicate connective tissue, and dashed arrows indicate bone dust (*lscc* lateral semicircular canal, *pscc* posterior semicircular canal, *PCW* posterior canal wall). Images taken with a fully digital surgical microscope (Munich Surgical Imaging MSI, Munich, Germany). Supplementary video: see QR code

The superior semicircular canal is accessed via a simple mastoidectomy with removal of the cortical bone and identification of the posterior wall of the external auditory canal, the sinus–dura angle, the lateral semicircular canal, the incus body, and the mastoid tegmen. Often, the middle fossa dura is not completely covered by bone, and small areas of the dura may need to be additionally exposed. The superior semicircular canal is then skeletonized using a fine diamond burr with exposure of the “blue line” (actually more of a “gray line”). After further thinning of the bone, punctiform openings of the semicircular canal are made anterior (as far away from the ampulla as possible) and posterior to the dehiscence. The remaining bone covering the membranous labyrinth at the small openings is lifted off with a 0.2-mm footplate hook (catalog number 224802, KARL STORZ, Tuttlingen, Germany). Small pieces of wet connective tissue are now placed first in the anterior opening (in the direction of the ampulla) and then in the posterior opening (in the direction of the common crus). Then bone dust is inserted into the semicircular canal toward the dehiscence (again, first in the anterior and then in the posterior opening). During the procedure, care must be taken to ensure that the semicircular canal is always covered with fluid. We use an irrigation fluid that is almost isotonic to perilymph (Eye-Lotion Balanced Salt Solution, Serumwerk Bernburg Vertriebs GmbH, Bernburg, Germany) for this and all other procedures involving opening of the inner ear. The suction tip, which should be as thin as possible, should not be held too close to the semicircular canal openings and at no time should fluid be aspirated directly from the opened semicircular canal. The superior semicircular canal and any exposed dura are then covered with bone pâté and TachoSil® (Takeda Pharma Vertrieb GmbH & Co., Berlin, Germany; Figs. 1 and 2; Video 1: see QR code and supplementary information). The wound is then closed using pericranial, subcutaneous, and skin sutures, and a controlled head pressure dressing is applied.

## Postoperative results

Both 35-year-old female patients presented with significant subjective impairment and symptoms typical of SCDS: abnormal perception of their own internal body sounds in the affected ear (e.g., eye movements, heartbeat, breathing, footsteps = autophony) as well as dizziness and unsteadiness when walking and standing triggered by movements, loud noises, or pressure changes (e.g., during Valsalva maneuvers or entering a tunnel, sometimes with a short-term deterioration in hearing). High-resolution computed tomography of the temporal bone revealed a dehiscence of the superior semicircular canal (Fig. 1).

**Fig. 3:**
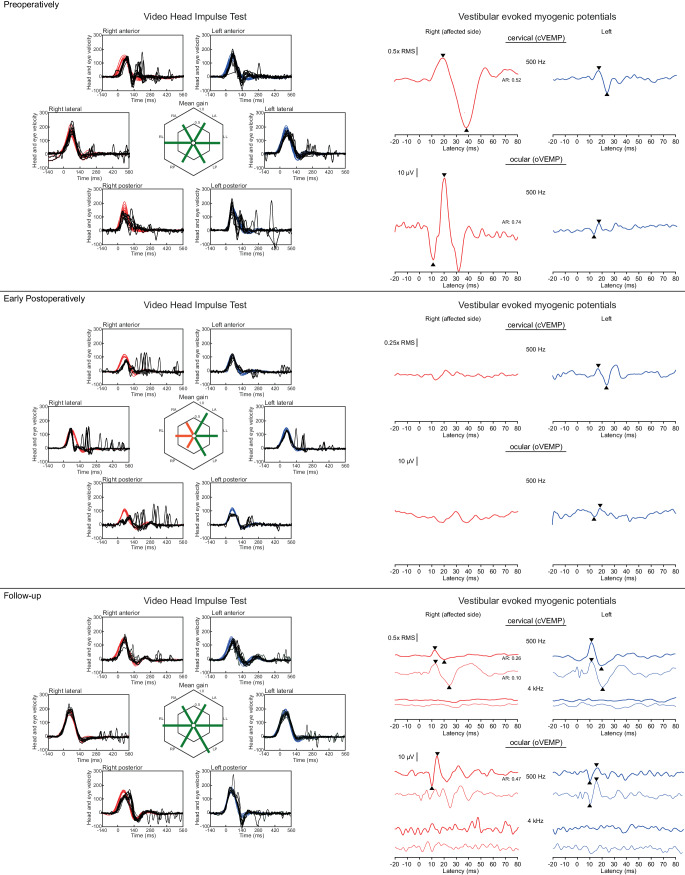
Vestibular evoked myogenic potentials (VEMPs) and video head impulse test results (vHIT) for patient 1. Preoperatively, the ratios of eye and head angular velocities (vHIT gain) were within normal range for all semicircular canals, but corrective saccades were visible for the affected right superior semicircular canal. After isolation of the dehiscence using transmastoid two-point plugging of the superior semicircular canal, the gain for all three semicircular canals on the right was initially significantly reduced and saccades occurred. In the long-term follow-up, the findings had approximated the preoperative situation, and corrective saccades hardly occurred or no longer occurred at all. Preoperatively, significantly increased cervical VEMP (*cVEMP*) and ocular VEMP (*oVEMP*) amplitudes were measurable at 500 Hz air conduction stimulation on the right compared with the contralateral left side. While no VEMP responses were present for the affected side early after surgery, the responses recovered and were similar to the contralateral amplitudes during long-term follow-up (thin lines indicate bone conduction stimulation on the mastoid; thick lines indicate air conduction stimulation). Postoperatively, as expected for a non-dehiscent labyrinth, no oVEMP signals could be evoked at 4 kHz air and bone conduction on either side

**Fig. 4:**
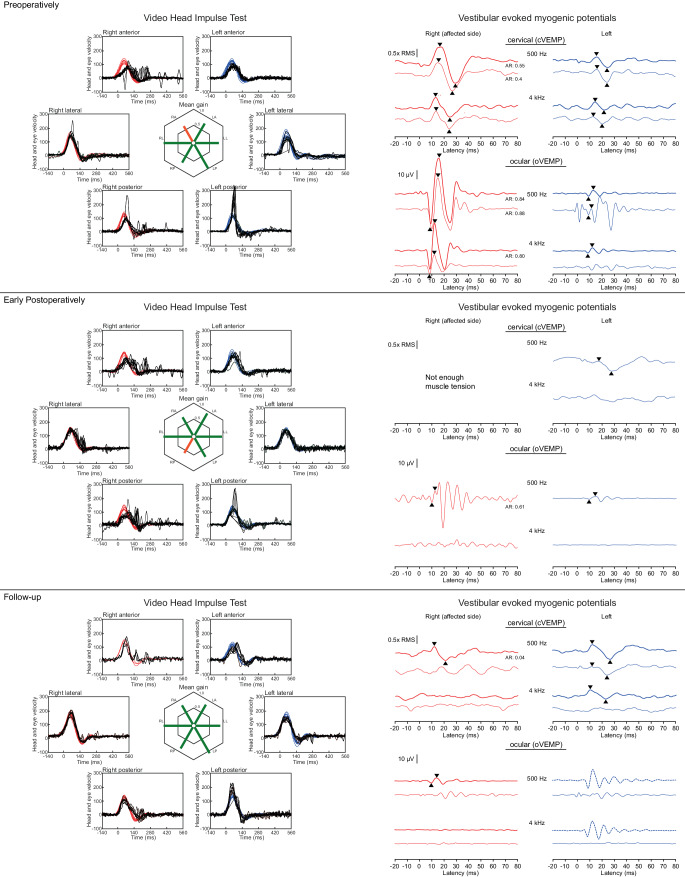
Vestibular evoked myogenic potentials (VEMPs) and video head impulse test results (vHIT) for patient 2. Preoperatively, the ratios of eye and head angular velocities (vHIT gain) for all semicircular canals (except for the affected right anterior semicircular canal) were within normal range. After isolation of the dehiscence using transmastoid two-point plugging of the superior semicircular canal, the gain for the right posterior and anterior semicircular canals was initially significantly reduced and corrective saccades occurred. In the long-term follow-up, the findings had approximated the preoperative situation and could be considered normal. Corrective saccades hardly occurred or no longer occurred at all. Preoperatively, significantly increased cervical VEMP (*cVEMP*) and ocular VEMP (*oVEMP*) amplitudes were measurable on the right side, and oVEMP responses were measurable also during stimulation with 4 kHz tone bursts. Postoperatively, oVEMPs were no longer measurable with 4 kHz stimulation, and the VEMP amplitudes at 500 Hz stimulation were equal on both sides. Normal cVEMPs and oVEMPs were measured in the long-term course. Thin lines indicate bone conduction stimulation; thick lines indicate air conduction stimulation.

Postoperatively, both patients experienced an almost complete regression of their symptoms indicating normalization of inner ear fluid dynamics. In addition, a reduction in the preoperatively pathologically increased amplitudes of VEMPs to 500 Hz stimulus frequency and absent ocular VEMPs (oVEMPs) to 4 kHz were observed over the long-term course of 1 year (Figs. 3 and 4; [4]). After initially (early postoperative) observable corrective saccades in the vHIT (Fig. 3b and 4b), the high-frequency VOR normalized in the long-term course (Fig. 3c and 4c). In patient 1, the vHIT gain for the superior semicircular canal was 0.7 preoperatively; 0.49, early postoperatively (day 4); and 0.75, long-term postoperatively (day 622). In patient 2, the vHIT gain was 0.64 preoperatively; 0.75, early postoperatively (day 2); and 0.79, long-term postoperatively (day 155). Pathological corrective saccades observed preoperatively in vHIT testing in the plane of the superior semicircular canal were not detectable or hardly detectable postoperatively.

## Discussion

Before surgical treatment of SCDS, a detailed patient consultation including explanation of the clinical picture and the underlying pathophysiology is essential, taking into account the individual severity of symptoms and the patient’s expectations. According to the consensus document of the Bárány Society, radiological dehiscence of the superior semicircular canal alone (i.e., SCD), which is often detected as an incidental finding on CT, does not constitute an adequate diagnosis and indication for surgical treatment. The diagnosis of a dehiscence *syndrome *of the superior semicircular canal (SCDS) should always be made in the context of the combination of symptoms, audiovestibular findings, and imaging according to the criteria of the ICVD [5, 21]. The decision on surgical treatment is made jointly in the sense of “shared decision-making” after careful and critical assessment of the nature/severity of the complaints, the audiovestibular test findings, and the patient’s expectations.

There is a relative lack of consensus in the literature on the optimal surgical technique. Surgical therapies include so-called recapping or resurfacing of the dehiscence, in the sense of an attempt to restore the continuity of the bony canal wall, or the so-called plugging (occlusion) of the dehiscence either via a middle fossa or a transmastoid approach [6, 8, 20]. In selected cases, round window reinforcement can also be considered—at least as an attempt at treatment. While the middle fossa approach is associated with higher invasiveness and the resurfacing or capping techniques are associated with higher failure rates, the advantages of isolating the dehiscence using transmastoid two-point plugging of the semicircular canal include avoidance of craniotomy/temporal lobe retraction, the familiarity of the approach for experienced ear surgeons, and the possibility of isolating the dehiscence without direct manipulation of the semicircular canal defect [1, 6, 13, 20, 22].

Contrary to the intuitive assumption that a complete interruption of the semicircular duct also leads to a loss of function of the receptor, both cases described here showed preservation of the high-frequency VOR (as measured by vHIT) in long-term follow-up.

One possible explanation can be found in the “flexible labyrinth model” according to Iversen and Rabbitt [9, 16] based on data from single-cell recordings of primary vestibular neurons after plugging of the horizontal semicircular canal in oyster fish (*Opsanus tau*; [15]). For application of the mathematical model to the human lateral semicircular canal [9], labyrinth morphology, endo- and perilymph fluid dynamics, and the deformability of the membranous labyrinth were taken into account. In summary, the model states that the VOR is reduced for *low-frequency* rotational accelerations after plugging the semicircular canal. For *higher-frequency* accelerations, this effect becomes progressively smaller. At frequencies greater than 5 Hz, the VOR gain is again close to the value for anatomically normal canals. A high-frequency acceleration of more than 5 Hz lies exactly within the operating range of the vHIT [3, 19]. The reason for frequency-dependent VOR preservation after canal plugging lies primarily in the deformability of the membranous labyrinth. At low-frequency accelerations, it behaves mainly rigidly. If the endolymph in the semicircular duct hits the obstacle, i.e., the plug, there is hardly any deformation of the membranous labyrinth. This means that no significant endolymph flow is possible in the area of the ampulla/cupula. For example, the VOR gain is reduced by a factor of 1000 at a frequency of 0.01 Hz (see Fig. 3c in Iversen and Rabbitt 2017, [9]). At frequencies greater than 5 Hz, however, the membranous labyrinth behaves elastically. If the endolymph now hits the obstacle, the semicircular duct deforms at this point (expansion) and opposite to it (contraction), resulting in a redistribution of endolymph within the remaining membranous labyrinth and thus in a deflection of the cupula.

The results in the oyster fish were confirmed in principle by measurements in other species, e.g., in rhesus monkeys [7], macaques [18], and squirrel monkeys [10]. Differences in the preservation of the high-frequency VOR between the individual species can be explained by different hydrodynamic properties of the labyrinths.

Preservation of high-frequency VOR function after plugging of the superior semicircular canal has also been observed for isolated cases in other studies [11, 17, 22], although the majority of studies dealing with plugging of the semicircular canal describe a deterioration in semicircular canal function.

## Practical conclusion

- Isolation of the dehiscence of the superior semicircular canal by transmastoid two-point plugging is an effective method of treating superior canal dehiscence syndrome.
- The high-frequency vestibulo-ocular reflex, as measured with video head impulse testing, can be preserved despite an interruption of the semicircular duct.

## Supplementary Information

**Video 1: **The video shows the surgical steps of isolating the dehiscence of the superior semicircular canal using transmastoid two-point semicircular canal plugging for a right ear (in English for German and English version of the article). Video recorded with a fully digital surgical microscope (Munich Surgical Imaging MSI, Munich, Germany).

## Notes

Except for the collection of routine measurement data, no studies on humans or animals were conducted for this article.

## Notes

The supplement containing this article is not sponsored by industry.
